# Exploring Observability of Attractor Cycles in Boolean Networks for Biomarker Detection

**DOI:** 10.1109/access.2019.2937133

**Published:** 2019-08-23

**Authors:** YUSHAN QIU, YULONG HUANG, SHAOBO TAN, LI DONGQI, ADA CHAELI VAN DER ZIJP-TAN, GLEN M. BORCHERT, HAO JIANG, JINGSHAN HUANG

**Affiliations:** 1 College of Mathematics and Statistics, Shenzhen University, Shenzhen 518000, China; 2 College of Allied Health Professions, University of South Alabama, Mobile, AL 36688, USA; 3 School of Computing, University of South Alabama, Mobile, AL 36688, USA; 4 College of Medicine, University of South Alabama, Mobile, AL 36688, USA; 5 School of Mathematics, Renmin University of China, Beijing 100872, China

**Keywords:** Attractor, Boolean networks, observability

## Abstract

Boolean Network (BN) is a simple and popular mathematical model that has attracted significant attention from systems biology due to its capacity to reveal genetic regulatory network behavior. In addition, observability, as an important network feature, plays a vital role in deciphering the underlying mechanisms driving a genetic regulatory network and has been widely investigated. Prior studies examined observability of BNs and other complex networks. That said, observability of attractor, which can serve as a biomarker for disease, has not been fully examined in the literature. In this study, we formulated a new definition for singleton or cyclic attractor observability in BNs and developed an effective methodology to resolve the captured problem. We also showed complexity is of *O*(*P^m^n*), when the maximal period of cyclic attractor is *P*, the number of attractor is *m* and the number of genes is *n*. Importantly, we have confirmed our method can faithfully predict the expression pattern of segment polarity genes in Drosophila melanogaster and showed it can effectively and efficiently deal with the captured observability problem.

## I. INTRODUCTION

Systems biology, refers to the study of biological systems component interactions and a field that has undergone rapid development in the post-genome era. Genetic regulatory networks (often simply referred to as genetic networks) have been intensively studied to better understand the interactions of various genes, molecules and proteins. Several formalisms have been employed to better model genetic regulatory processes. BNs [1]-[3] have received particular attention owing to their capacity to capture the highly dynamic behavior of genetic regulations.

The first interesting problem typically encountered is Boolean network topological structure, such as fixed points, cycles and attractors basin, etc [4]-[6]. Many studies dealing with the analysis of attractors of randomly given BNs have been performed [3], [7] in large part due to the fact that different attractors can be utilized to represent unique cell types. Several methods have been suggested [8]-[10] to enumerate and/or identify attractors in BNs. As examples, Devloo *et al.* proposed an effective method based on transforming to a constraint satisfaction problem [8], and Irons developed another method employing small subnetworks [9]. Zhang *et al.* [10] proposed algorithms to enumerate singleton and small attractors, and also, analyzed the time complexities of the average cases of these algorithms. Of note, it has been shown that singleton attractors (i.e., a fixed state) detection problem is NP-hard [11]. In addition, Akutsu *et al.* [12] developed algorithms with guaranteed “worst case” time complexity allowing singleton attractor detection in BNs of limited Boolean functions. Importantly, all these approaches dealt specifically with 2^*n*^×2^*n*^ matrices (where *n* represents the gene number in a BN), making applying them to large-scale BNs exceedingly challenging. Therefore, Akutsu *et al.* [13] developed an integer linear programming (ILP-based) approach to construct the attractor control problem of medium size BNs. Even though the authors in [13] have reported that the attractor control problem is $\sum_{2}^{P}$-hard, an ILP-based methodology can be applied to moderate size BNs. Each of these studies, however, has simply examined randomly generated, simple BNs (i.e., one cell type). It is therefore critically necessary to develop strategies capable of analyzing multiple BNs (i.e., various cell types). In the genuine cellular context, there are various kinds of cells, and it is therefore simply more realistic to perform attractor analysis for multiple BNs. Motivated by this, Qiu *et al.* [14] recently examined this problem and employed the ILP-based method to solve it.

The control of Boolean networks is also a challenging problem. According to control theory, dynamic systems are controllable if they can be driven from any initial state to any desired final state with a suitable choice of inputs [15]. Several studies have been conducted on this problem [16], [17], and when a random Boolean network is examined, the principle interest lies on the stationary distribution of the system. The reachability problem as in control theory becomes a common concern for the deterministic network [18].

Observability, as a dual of controllability, is a significant concept which needs further exploration. Several studies have recently examined such observability problems. Cheng and Qi [19], [20] adopted a semi-tensor strategy to examine observability and related problems. While their design was effective for formulating and constructing the problems, the application of their method to large-scale BNs is also impractical. Of note, Liu *et al.* [21] proposed a novel view of complex networks observability. They showed the aim of attractors observability was to determine the minimum consecutive nodes necessary to discriminate distinct attractor cycles from each other in the system. This work provided a great opportunity for one to diagnose disease types since different attractors represent different cell types. That said, we suggest here that the problem proposed represents a particular case, binary alphabet of the minimal key problem examined in [12], [22], [23]. It has been showed that the minimal key problem is NP-hard even when dealing with the binary alphabet [23]. Cheng *et al.* [24] have proposed to apply the ILP-based method to solve the observability problem which was limited to singleton attractors. However, cyclic attractors exist in the real-world and the issue of observability for cyclic attractors has not yet been fully addressed. Qiu *et al.* [25] have proposed a method to study the observability of singleton and cyclic attractor. However, the analysis on the sensitivity of number of genes and attractors need to be further explored. In addition, the application of our method to the real model requires further discussion. Thus, the problem we try to address in this study is to identify the minimum set of contiguous nodes such that we can discriminate the singleton or cyclic attractors. Furthermore, we analyze the distribution of gene number and attractor number for distinguishing the cell types, respectively. We show that our proposed method is efficient and can effectively solve large-size problems in *O*(*P^m^n*) for the worst case, where *P* is the maximum number of period of cyclic attractor, *m* is the number of (singleton or cyclic) attractors and *n* is the number of genes (nodes) in a BN.

This paper has two main contributions. First, we formulate a new biological problem based on Boolean networks to discriminate the cell type with minimum consecutive nodes. Second, we propose an effective and efficient method, (works in *O*(*P^m^n*)) to solve the captured problem. By integrating the expression pattern of the segment polarity genes in Drosophila melanogaster, we have demonstrated that our approach can efficiently identify the minimum contiguous genes or proteins to discriminate the attractors. Furthermore, experimentation confirms that our proposed methodology is extremely efficient and can effectively solve the problem in seconds.

The remainder of the paper is structured as follows. In the “Problem formulation” section, we introduce some background and formulate the problem. “Methodology” presents a novel method for our captured problem and give two propositions. “Numerical Experiments” describes the materials and experimentation results to validate the efficiency and effectiveness of our method. And finally, conclusions and future directions are described in the last section.

## II. PROBLEM FORMULATION

This section (1) introduces the BN model and the attractor detection problem which are closely related to attractor observability, and (2) then formulates the attractor observability problem in BNs.

A BN *G*(*V, F*) consists of a set of nodes *V* used to represent multiple genes *V* = {*ν*_1_, *ν*_2_, ⋯ , *ν_n_*} and a set of Boolean functions *F* = (*f*_1_, *f*_2_, ⋯ , *f_n_*) where *f_i_* : {0, 1}^*n*^ → {0, 1}. Each node (e.g., a gene) is assumed to take either 1 (active) or 0 (inactive) as its state value. And *ν_i_*(*t*) is denoted as the state of node *i* at time *t*, after which the state of node *i* at time *t* + 1 is given as follow:

$$v_{i}(t+1)=f_{i}(v(t)),\;i=1,2,\ldots ,n$$

here

$$v(t)=[v_{i_{1}}(t),\cdots ,v_{i_{h_{i}}}(t)].$$

This indicates the gene state *ν_i_* at time *t* + 1 is determined by the *h_i_* gene states at previous time *t*, where *h_i_* represents input node number called *indegree* of *ν_i_*. Moreover, the maximum indegree of BN is denoted by *H* = *max_i_*{*h_i_*}. We note *x* ∨ *y*, *x* ∧ *y*, ¬*x*, *x* ⊕ *y* is logical OR of *x* and *y*, logical AND of *x* and *y*, logical NOT of *x* and exclusive OR of *x* and *y*, respectively. Overall gene expressions at time *t* in a BN is determined by

$$gap(t)=[v_{1}(t),v_{2}(t),\ldots ,v_{n}(t)]$$

and called the network Gene Activity Profile (GAP) at time *t*. Note that gap(*t*) ranges from [0, 0, . . . , 0] to [1, 1, . . . , 1], therefore we have a total of 2^*n*^ possible global states. The following details an example of a BN.

*Example 1:*

{v1(t+1)=¬v2(t),v2(t+1)=¬v1(t)∨v3(t),v3(t+1)=v1(t).}

**FIGURE 1.**
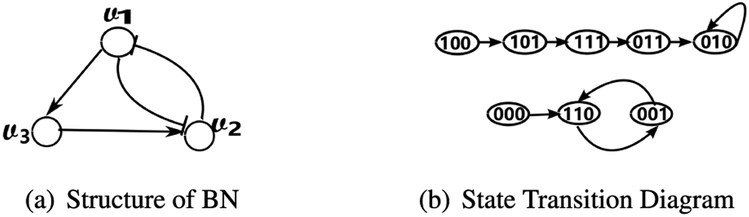
Example of a BN.

Each gene *ν_i_* is regulated by a Boolean function *f_i_*. A state transition diagram and the dynamics of a BN are shown in Figure 1. The BN truth table is showed in Table 1. For example, the fourth row of the table shows that if the state of BN is [0, 1, 1] at time *t*, then the state at time *t* + 1 will be [0, 1, 0]. Similarly, the arc from 011 to 010 indicates that when the BN state is [0, 1, 1] at time *t*, the state is [0, 1, 0] at time *t* + 1. In total, there are 2^*n*^ potential states in a BN containing *n* nodes. Thus, the state transition table contains 2^*n*^ rows, and the corresponding state transition diagram has 2^*n*^ vertices.

The gene state is determined by its associated regulatory function. Given the initial state of a network, a BN will ultimately enter into a certain set of global states (i.e., a directed cycle as depicted in state transition diagram). We denote such a set as an attractor. When an attractor corresponds to one global state (i.e., a single fixed point), it is classified as a singleton attractor. In all other events, it would be classified as a cyclic attractor, and we denote *p* as the period of cyclic attractor if it consists of *p* global states. We can see from Figure 1 that the network will eventually evolve into one of two attractors: either a singleton attractor, [0, 1, 0] or a cyclic attractor of period 2, [1, 1, 0] ↔ [0, 0, 1].

### A. OBSERVABILITY OF ATTRACTORS

Once attractors are detected, one can conduct an analysis of the BN attractor control problem [13], [14]. Observability, as a compliment of controllability, has been examined by Cheng and Qi [19]. They developed necessary and sufficient conditions for dealing with the captured observability problem. Cheng *et al.* [24] proposed an efficient and effective approach for solving singleton attractor observability in BNs. That said, the problem of observability for cyclic attractor has not yet been resolved. Thus, in this study, we propose a new methodology for addressing the observability problem of (singleton and cyclic) attractors, not restricted to singleton ones in BNs, and therefore representative of the realities of biology. Importantly, when a BN is given, attractors can be further detected. In brief, assume a set of singleton or cyclic attractors is given as *S* = {*S*_1_, *S*_2_, . . . , *S_m_*}, our method then attempts to identify a tandem gene set of minimum cardinality that can be used to distinguish distinct attractor cycles from each other in a system. For instance, we consider four singleton or cyclic attractors with seven genes which is given as *S* = {*S*_1_, *S*_2_, *S*_3_, *S*_4_}.

$$S_{1}=\{(0,0,1,1,0,1,1)\};\;S_{2}=\{(1,1,0,0,0,1,0)\};\;S_{3}=\{(1,1,0,1,0,1,0),(0,0,1,1,1,0,0)\};\;S_{4}=\{(0,0,1,0,0,1,1),(1,1,0,1,1,1,0)\}.$$

where *S*_1_ and *S*_2_ are singleton attractors, *S*_3_ and *S*_4_ correspond to cyclic attractors of period 2. Note that there are four attractors in total, it would require at least two nodes to distinguish them. Next, if we observe the third and forth nodes only, we can identify which attractor a system belongs to (i.e., (1, 1), (0, 0), (0, 1), (1, 0) mean *S*_1_, *S*_2_, *S*_3_ and *S*_4_, respectively). As such, the attractor observability problem is formulated as:

*Definition* 1: ATTRACTOR OBSERVABILITY (AO)

Instance: List of (singleton or cyclic) attractors in a BN,

Problem: Determine the minimum cardinality of consecutive nodes that can distinguish different attractor cycles from one another in a BN.

## III. METHODOLOGY

In the following section, we describe our attractor observability problem approach.

### A. PROPOSED ALGORITHM FOR AO

Assume a set of (singleton or cyclic) attractors are given as *S* = {*S*_1_, *S*_2_, . . . , *S_m_*}, where size of each *S_i_*, (*i* ∈ {1, 2, ⋯ , *m*}) is *l_i_*. We then have total of *L* = *l*_1_ ∙ *l*_2_ ⋯ *l_N_* possible combinations and employ a matrix set *A* = {*A*_1_, *A*_2_, ⋯ , *A_L_*} to describe all possible case combinations. Each matrix *A_i_*, *i* ∈ {1, 2, ⋯ , *L*} is therefore of size *m* × *n* (*m*: attractor number; *n*: nodes (genes) number). Then an individual row *A_i_* corresponds to one global state from singleton attractor or cyclic attractor and individual columns represent gene states. We then develop our algorithm based on matrix *A* for AO. The major steps (procedures) for our algorithm are details below.

#### The Procedure

**Step I**: For *i* ∈ {1, 2, ⋯ , *L*}, assume the size of matrix *A_i_* is *m* × *n*, where *m* is attractor number and *n* is gene number. Repeat Step II-VIII.

**Step II**: Apply arithmetic ⊕ operator for *A_i_* to generate a new matrix *biState_i_* which is of size $C_{m}^{2}\times n$. We then define

$$\mathrm{biState}(t,j)=A(i,j)⊕A(k,j),\;\;t=(m-\frac{i}{2})(i-1)+k-i.\;\;1\leq i<k\leq m,\;1\leq j\leq n,\;i,j,k,t\in N.$$

recall that the arithmetic ⊕ is presented as follows:

x⊕y=(x∧¬y)∨(¬x∧y).

**Step III**: For an indiviual row $t\;\epsilon \;C_{m}^{2}$ in matrix *biState_i_*, identify consecutive 0′*s* with length no less than ⌈log_2_(*m*)⌉ and place them in matrix *B_con_* (assume that total entry number in *B*_*con*_ is *r*). Another matrix *B*_*index*_ size *r* × 2 is then created to record indices of *B_con_* (e.g., *B_index_*(*i*, 1) and *B_index_*(*i*, 2) correspond to the first and last elements of row *i* of *B_con_*, *i* ∈ {1, ⋯ , *r*}, respectively).

**Step IV**: Generate the union of *B*_*con*_ and denote it as *B*_*uni*_.

**Step V**: Let *U* represent a vector which ranges from 1 to *n* (i.e., *U* = {1, ⋯ , *n*}). If *U* \ *B*_*uni*_ ≠ ∅ holds, then we can conclude this represents the desired minimal contiguous nodes and corresponding minimal number for *A*_*i*_ is ⌈log_2_(*m*)⌉ and denoted as *minL_i_*. Therefore, the desired minimal number of consecutive nodes has been identified (i.e., ⌈log_2_(*m*)⌉ and stop. Otherwise, continue.

**Step VI**: Employ a sorting algorithm (such as bucket sort) to *B*_*index*_. Then rank vector *B*_*index*_ (:, 1) in an ascending order and update the order in *B*_*index*_(:, 2) accordingly. As an example,

(1) $$B_{\mathrm{index}}=\left[\begin{array}{cc} 1 & 5\\ 4 & 7\\ 3 & 9\\ 3 & 6\\ 2 & 8 \end{array}\right].$$

after applying the sorting algorithm, it becomes

(2) $$B_{\mathrm{index}}=\left[\begin{array}{cc} 1 & 5\\ 2 & 8\\ 3 & 6\\ 3 & 9\\ 4 & 7 \end{array}\right].$$

**Step VII**: Compare last elements of rows with identical first element in *B*_*index*_. Discard rows with smaller last elements and keep the rows with the largest last element in a new matrix *B_ni_* of size *l* × 2 as the rows with smaller last elements are subsets of the row with the largest last element. Thus, *B_ni_* is

(3) $$B_{\mathrm{ni}}=\left[\begin{array}{cc} 1 & 5\\ 2 & 8\\ 3 & 9\\ 4 & 7 \end{array}\right].$$

**Step VIII**: The desired node number for *A_i_* is

$$\max\{\min\{B_{\mathrm{ni}}(j,2)-B_{ni}(j+1,1)+3\},\lceil \log_{2}(m)\rceil \},\;\;j\in \{1,\cdots ,l-1\}.$$

and denoted as *minL_i_*. If *minL_i_* = ⌈log_2_(*m*)⌉, stop algorithm. Otherwise, return to Step II.

**Sep IX**: Compare *minL_i_*, *i* ∈ {1, 2, ⋯ , *L*} value and choose the minimum value as the desired minimal number of consecutive nodes necessary to distinguish different attractors.

*Proposition 1:* If element number in *B_uni_* from matrix *A_i_*, *i* ∈ {1, 2, ⋯ , *L*} is less than *n*, then the minimal number of desired nodes for *A_i_*, *i* ∈ {1, 2, ⋯ , *L*} equals to ⌈log_2_(*m*)⌉.

*Proof:* Let (*ν*_1_, *ν*_2_, . . . , *ν_n_*) represent *n* consecutive nodes in a given BN. Assume that element number in *B*_*uni*_ < *n*, at least one element in $B_{uni}^{c}$ must exist which is denoted as *ν_x_*. Then it suffices to show that there exists a pair of (*x*_1_, *x*_2_), such that (*ν*_*x*_1__ , . . . , *ν_x_*, . . . , *ν*_*x*_2__) represent desired minimal consecutive nodes, where *x*_2_−*x*_1_ + 1 = ⌈log_2_(*m*)⌉. In other words, we must show that there is no row with elements that are all 0 from column *x*_1_ to *x*_2_ in the matrix *biState*. For *x*-th column elements, there are two optional values (i.e., 0 or 1). One possible case is *biState*_*tx*_ = 0 (for some *t*), where the length of consecutive 0’s covering *biState*_*tx*_ is < ⌈log_2_(*m*)⌉. Otherwise, *x* ∈ *B*_*uni*_. Therefore, this implies that no less than one 1 exists from column *x*_1_ to *x*_2_. As another possible case, i.e., *biState_ik_* = 1, then it is obvious that no continuous 0’s can exist from column *x*_1_ to *x*_2_. Based on this, we conclude that (*ν*_*x*_1__ , . . . , *ν_x_*, . . . , *ν*_*x*_2__) is the desired minimal consecutive nodes for one attractor combination *A_i_* in the BN. □

*Proposition 2:* If *B_uni_* element number from matrix *A_i_*, *i* ∈ {1, 2, ⋯ , *L*} is *n*, then the minimal cardinality of desired nodes for *A_i_*, *i* ∈ {1, 2, ⋯ , *L*} is:

$$\max\{\min\{B_{\mathrm{ni}}(j,2)-B_{ni}(j+1,1)+3\},\lceil \log_{2}(m)\rceil \},$$

where *j* ∈ {1, ⋯ , *l* − 1}.

*Proof:* Consider two following cases below:

*Case one:* If

$$\min\{B_{\mathrm{ni}}(j,2)-B_{ni}(j+1,1)+3\}\geq \lceil \log_{2}(m)\rceil$$

then it suffices to demonstrate that in the sub-matrix *biState*(:, *B_ni_*(*j* + 1, 1) − 1 : *B_ni_*(*j*, 2) + 1), there is no row with all 0 elements. Note that *biState*_*t*_1_,*B_ni_*(*j*,2)_ corresponds to the last 0 in the *t*_1_ row starting from column *B_ni_*(*j*, 1) to *B_ni_*(*j*, 2) (for some *t*_1_), indicating that *biState*_*t*_1_,*B_ni_*(*j*,2)+1_ value must be 1. However, if *t* ≠ *t*_1_, there are then two potential values of *biState*_*t,B_ni_*(*j*,2)+1_ (i.e., 0 or 1).

In the case of *biState*_*t,B_ni_*(*j*,2)+1_ = 0, it implies that *biState*_*t,B_ni_*(*j*+1,1)−1_ must take 1. Otherwise, *B_ni_*(*j* + 1, 1) is replaced by *B_ni_*(*j* + 1, 1) − 1.

In the case of *biState*_*t,B_ni_*(*j*,2)+1_ = 1, it has been found that in row *t*, no less than one 1 exists within column *B_ni_*(*j*+1, 1)−1 to *B_ni_*(*j*, 2) + 1.

Similarly, we note that *biState*_*t*_2_,*B_ni_*(*j*+1,1)_ is the first 0 in the *t*_2_ row starting from column *B_ni_*(*j* + 1, 1) to *B_ni_*(*j* + 1, 2) (for some *t*_2_), this implies that *biState*_*t*_2_,*B_ni_*(*j*+1,1)−1_ = 1.

If *t*_3_ ≠ *t*_2_, *biState*_*t*_3_,*B_ni_*(*j*+1,1)−1_ may take 0 or 1.

If *biState*_*t*_3_,*B_ni_*(*j*+1,1)−1_ = 0, then obviously the length of consecutive 0’s containing *biState*_*t*_3_,*B_ni_*(*j*+1,1)−1_ in row *t*_3_ is < ⌈log_2_(*m*)⌉. Otherwise, *B_ni_*(*j* + 1, 1) will not be the original one.

If *biState*_*t*_3_,*B_ni_*(*j*+1,1)−1_ = 1, then it is obvious that no less than one 1 exists within column *B_ni_*(*j* + 1, 1) − 1 to *B_ni_*(*j*, 2) + 1 for row *t*_3_. Thus, min{*B_ni_*(*j*, 2) − *B_ni_*(*j* + 1, 1) + 3} gives the solution.

*Case two:* If

$$\min\{B_{\mathrm{ni}}(j,2)-B_{ni}(j+1,1)+3\}<\lceil \log_{2}(m)\rceil$$

then it is sufficient to consider this case (i.e., *biState*_*t*_3_,*B_ni_*(*j*+1,1)−1_ is 0), due to the fact that for the other cases, min{*B_ni_*(*j*, 2) − *B_ni_*(*j* + 1, 1) + 3} is not less than ⌈log_2_(*m*)⌉. Thus, we extend indicated columns to guarantee containing column *biState*_*t*_3_,*B_ni_*(*j*+1,1)−1_ and also guarantee the length of them is ⌈log_2_(*m*)⌉. Then columns of length ⌈log_2_(*m*)⌉ will produce the desired solution. Assume

$$c=\max\{\min\{B_{\mathrm{ni}}(j,2)-B_{ni}(j+1,1)+3\},\lceil \log_{2}(m)\rceil \},\;\;j\in \{1,\cdots ,l-1\}$$

and since we have shown there exist node set that can differentiate different cell types, we can next prove *c* represents the optimal solution. Put another way, there is no set of nodes length *c′* (*c′* < *c*) that can distinguish *A*_*i*_ attractors. Assume there exist a column set (*c*_1_, ⋯ , *c*_*t*_) with length *c′*, then it is sufficient to show that *c′* < *c* does not hold. If *c′* is less than *c*, then a row exists such that the set of column from column *c*_1_ to *c_t_* exactly represents a subset of column *B_ni_*(*j*, 1) to *B_ni_*(*j*, 2). This means that a row exists with elements all corresponding to 0’s. Therefore, we can conclude that *c′* does not exist such that *c′* < *c*, and max {min{*B_ni_*(*j*, 2) − *B_ni_*(*j* + 1, 1) + 3}, ⌈log_2_(*m*)⌉}, *j* ∈ {1, ⋯ , *l* − 1} corresponds to the desired solution of *A_i_*. □

We give an example to illustrate the first case of our algorithm.

*Example 2:* Let *A* be given below:

(4) $$A=\left[\begin{array}{cccccc} 0 & 1 & 0 & 1 & 0 & 1\\ 1 & 0 & 1 & 0 & 0 & 0\\ 0 & 1 & 0 & 0 & 1 & 0\\ 1 & 1 & 1 & 1 & 1 & 0 \end{array}\right].$$

Then we have

(5) $$\mathrm{biState}=\left[\begin{array}{cccccc} 1 & 1 & 1 & 1 & 0 & 1\\ 0 & 0 & 0 & 1 & 1 & 1\\ 1 & 0 & 1 & 0 & 1 & 1\\ 1 & 1 & 1 & 0 & 1 & 0\\ 0 & 1 & 0 & 1 & 1 & 0\\ 1 & 0 & 1 & 1 & 0 & 0 \end{array}\right].$$

and we can see that the matrix *B_con_* will be in the form

(6) $$B_{con}=\{[1\;2\;3],[5\;6]\}.$$

It is noted that the union of *B*_*con*_ is {[1 2 3], [5 6]} which is a subset of *U*. Thus any subset of the remaining elements of *U* \ *B*_*con*_ which is of length ⌈log_2_(*m*)⌉ (i.e., 2) will be the desired set of nodes. Actually, the elements of *U* \ *B*_*con*_ is 4, all the subset including 4 of length 2 is [3, 4] or [4, 5] which are exactly the desired minimal set of nodes necessary to discriminate attractors. Thus, [*ν*_3_, *ν*_4_] or [*ν*_4_, *ν*_5_] may be taken to represent the desired minimal consecutive nodes. However, if the union of *B*_*con*_ is exactly equal to *U*, for instance,

(7) $$A=\left[\begin{array}{cccccc} 1 & 0 & 0 & 1 & 0 & 1\\ 0 & 1 & 1 & 0 & 1 & 1\\ 1 & 0 & 1 & 0 & 1 & 0\\ 0 & 1 & 0 & 0 & 0 & 1 \end{array}\right].$$

Then we have

(8) $$\mathrm{biState}=\left[\begin{array}{cccccc} 1 & 1 & 1 & 1 & 1 & 0\\ 0 & 0 & 1 & 1 & 1 & 1\\ 1 & 1 & 0 & 1 & 0 & 0\\ 1 & 1 & 0 & 0 & 0 & 1\\ 0 & 0 & 1 & 0 & 1 & 0\\ 1 & 1 & 1 & 0 & 1 & 1 \end{array}\right].$$

Similarly, we can obtain the matrix *B*_*con*_ which is in this form:

$$B_{con}=\{[1\;2],[5\;6],[3\;4\;5],[1\;2]\}.$$

Since the union of vectors in *B_con_* ranges from 1 to *n*, it is necessary to apply Step VI. Thus, the matrix *B*_*index*_ is sorted using bucket sort algorithm according to the first column and keep the row with the greatest last element in *B_index_*, then *B*_*ni*_ is given by

(9) $$B_{ni}=\left[\begin{array}{cc} 1 & 2\\ 3 & 5\\ 5 & 6 \end{array}\right].$$

Then applying our proposed algorithm, the minimum set of columns is as follows:

$$\max\left\{\min_{j\in \{1,\cdots ,l-1\}}\{B_{ni}(j,2)-B_{ni}(j+1,1)\}+3,\lceil \log\;2(m)\rceil \right\}$$

which is equal to 2. Furthermore, since the row number of *A* is 4, it requires at least ⌈log_2_(*m*)⌉ (i.e., 2) to distinguish the attractors, and this implies that it is impossible to find any less number for the other combination of attractor set. Thus, we conclude the length of the minimal set of nodes to distinguish the attractors is two. Based on this example, (*ν*_2_, *ν*_3_) likely represents the desired minimal consecutive nodes.

In the case where a BN consists of singleton and cyclic attractors, we consider the following example to illustrate our method.

*Example 3:* We assume the steady states are as follows,

$$S_{1}=\{(0,1,0,1,1,0)\};\;\;S_{2}=\{(0,0,1,0,1,0)\};\;S_{3}=\{(0,0,1,0,1,1),(1,1,0,1,0,1)\};\;S_{4}=\{(0,1,0,0,1,0),(1,0,1,0,1,0)\}.$$

then there are totally *l*_1_ ∙ *l*_2_ ∙ *l*_3_ ∙ *l*_4_ = 4 combinations. It is easy to see that the combination can be

(10) $$A_{1}=\left[\begin{array}{cccccc} 0 & 1 & 0 & 1 & 1 & 0\\ 0 & 0 & 1 & 0 & 1 & 0\\ 0 & 0 & 1 & 0 & 1 & 1\\ 0 & 1 & 0 & 0 & 1 & 0 \end{array}\right];$$

(11) $$A_{2}=\left[\begin{array}{cccccc} 0 & 1 & 0 & 1 & 1 & 0\\ 0 & 0 & 1 & 0 & 1 & 0\\ 0 & 0 & 1 & 0 & 1 & 1\\ 1 & 0 & 1 & 0 & 1 & 0 \end{array}\right];$$

(12) $$A_{3}=\left[\begin{array}{cccccc} 0 & 1 & 0 & 1 & 1 & 0\\ 0 & 0 & 1 & 0 & 1 & 0\\ 1 & 1 & 0 & 1 & 0 & 1\\ 0 & 1 & 0 & 0 & 1 & 0 \end{array}\right];$$

(13) $$A_{4}=\left[\begin{array}{cccccc} 0 & 1 & 0 & 1 & 1 & 0\\ 0 & 0 & 1 & 0 & 1 & 0\\ 1 & 1 & 0 & 1 & 0 & 1\\ 1 & 0 & 1 & 0 & 1 & 0 \end{array}\right].$$

By applying our algorithm, the minimal length of consecutive nodes for each combination *A_i_*(*i* = 1, 2, 3, 4) are 4, 6, 3 and 2, respectively. After comparing the above values, we conclude that the minimal cardinality of consecutive nodes necessary to distinguish different attractor cycles is 2. And the cyclic attractors are of period 2, thus the algorithm efficiently works in *O*(2*^m^n*). Furthermore, it shall be noted that *m* is typically extremely small, and it is therefore reasonable to utilize our method to singleton or cyclic attractors with large-scale networks.

### B. COMPLEXITY ANALYSIS

We have conducted a complexity analysis for our algorithm. The principle computational costs come from two parts: one comes from generating the matrix *biState* and the other comes from the sorting algorithm. The first part clearly requires $O(C_{m}^{2}n)$ operations where $C_{2}^{m}=m(m-1)∕2$. Note that the number of attractors in a given BN is usually quite small, and as such conclude the computational complexity of this step is *O*(*n*). For the sorting algorithm in Step VI, we have adopted bucket sort that works in *O*(*n*), and repeat the algorithm for *P^m^* time such that all possible combinations of singleton or cyclic attractors are considered. Thus, we conclude our algorithm is both effective and efficient for the problem of attractor observability and works in *O*(*P^m^n*).

## IV. NUMERICAL EXPERIMENTS

In this section, we performed computational experiments to validate our methodoloy for attractor observability. Initially, we randomly generated a singleton attractor set and repeated the experiment ten times with different simulated attractors. Then we took the average value as the final result. Notations utilized are as follows.

- *m*: attractor number;
- *n*: node number;
- time: average time (in seconds) for each trial;
- numNode: the desired minimal node number.

We utilize the above notations herein.

As seen in Table 2 our method was effective and efficient in solving the observability problem of singleton attractors for large-scale networks. Although the size of BN was up to 300 nodes, the average elapsed time was less than one second which is much faster than the ILP-based method [24]. Besides, the desired number of nodes to distinguish different attractor cycles was consistently small although the node number was large. For our next analysis, we randomly generated a cyclic attractor set, and set the period of cyclic attractors to two. Computational times are shown in Table 3 which illustrated the efficiency of our method. Running time was around 1 second even the number of network nodes was set to 1000. Therefore, our algorithm can be applied to cyclic attractors, instead of being confined to singleton attractors.

**FIGURE 2.**
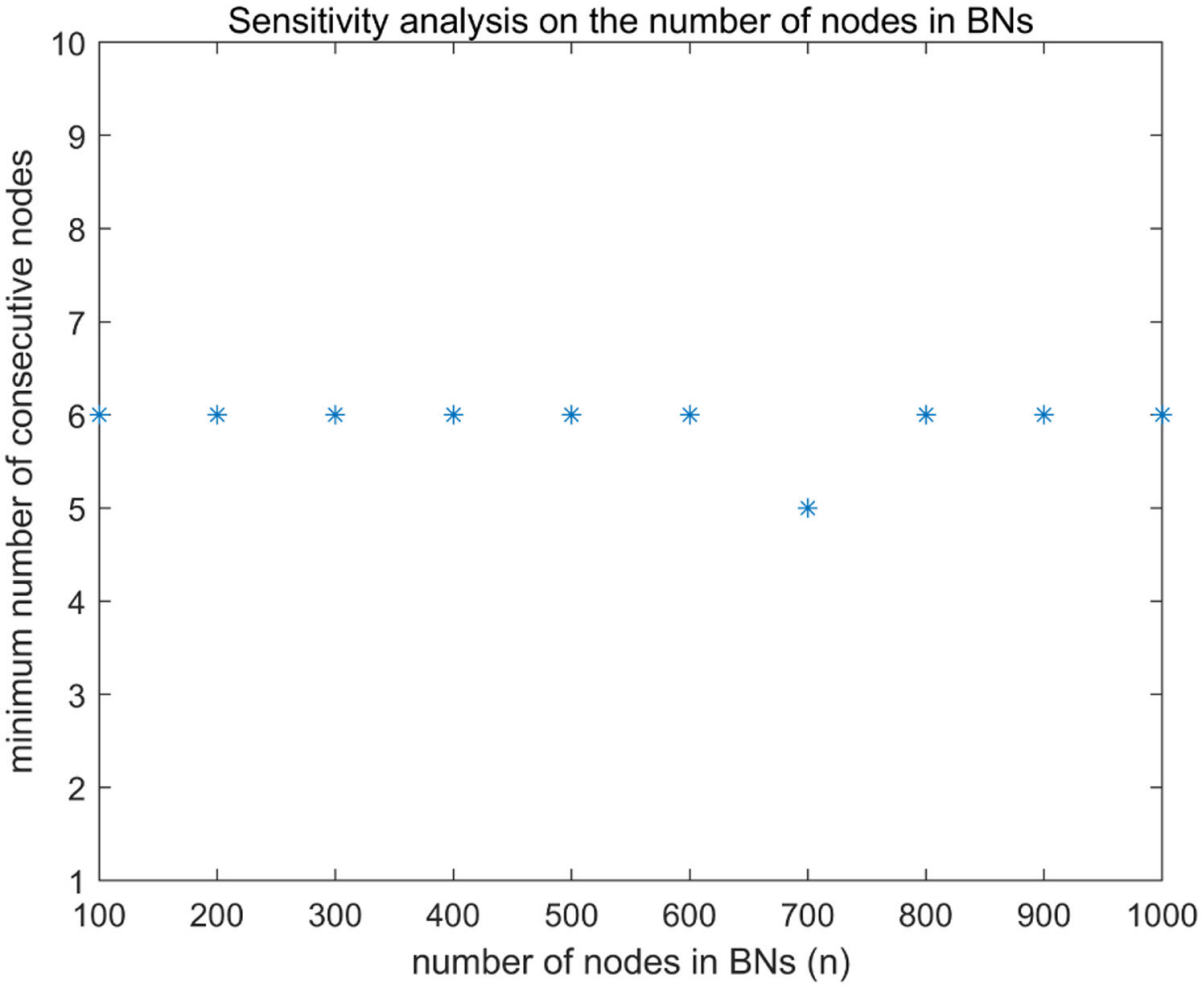
Sensitivity analysis on the number of nodes in BNs. Step size 100 is used for *n* in [100,1000] and *m* is fixed to be 20.

To examine the distribution of the desired number of nodes when the number of attractor was fixed (*m*), we fixed *m* as 20 and conducted analysis for different numbers of *n* ranging from 100 to 1000. The desired number of nodes are shown in Figure 2. Since most of the desired numbers are 6, this indicates that our method is insensitive to the node number in BNs with a fixed number of attractors. Therefore, *n* variation does not significantly effect desired node number for discriminating the attractor system.

**FIGURE 3.**
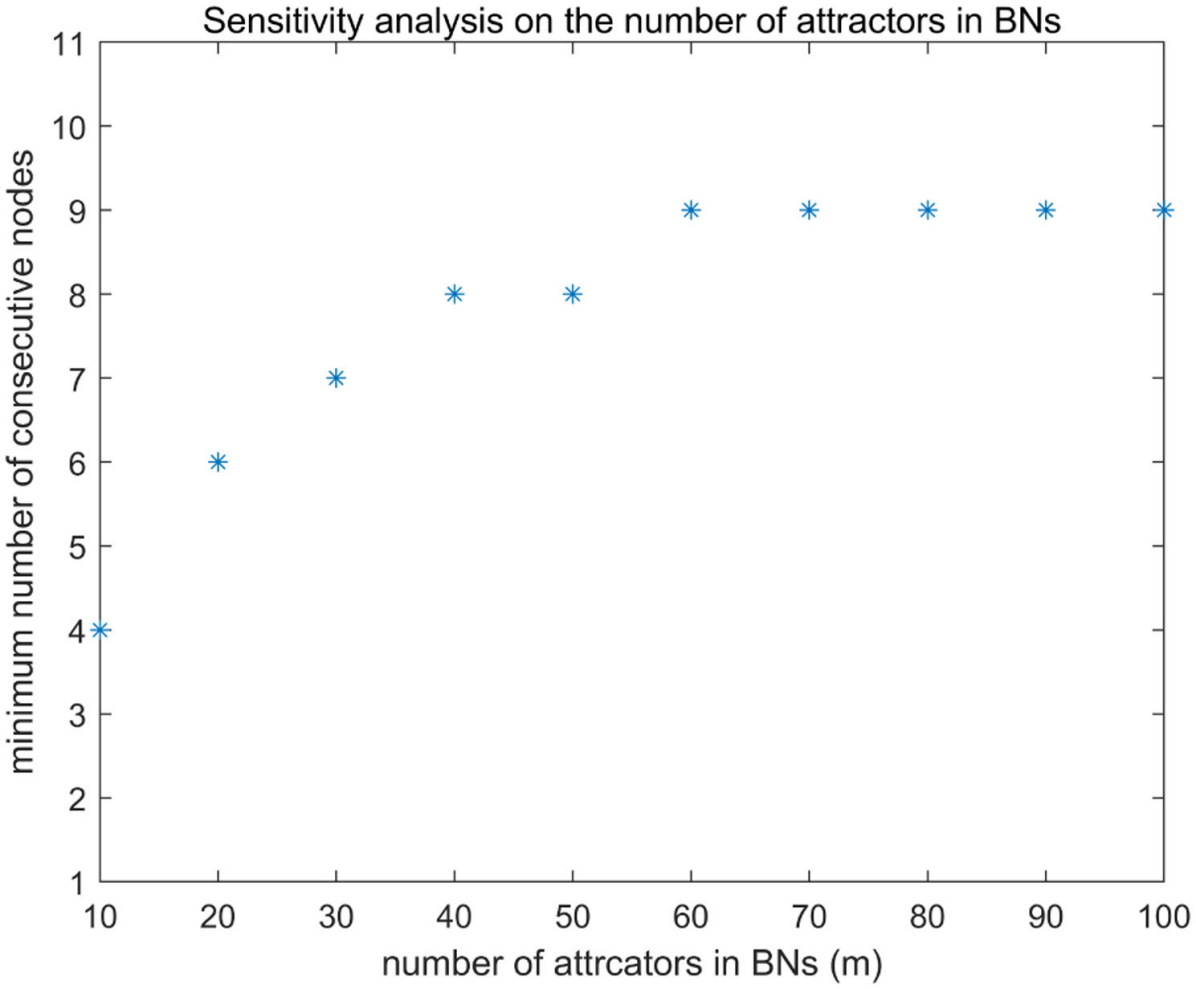
Sensitivity analysis on the number of attractors in BNs. Step size 10 is used for *m* in [10,100] and *n* is fixed to be 100.

We also examined the distribution of the desired node number when the number of nodes (*n*) was fixed to be 100. We adopted 10 as the step size for *m* in [10,100]. As illustrated in Figure 3, most of the desired numbers of consecutive nodes are quite small although *m* is large. Therefore, we can discriminate the set of attractors in large-scale network by identifying the small list of desired nodes.

To further verify our strategy, we applied our methodology to a popular genetic regulatory model in Drosophila melanogaster. Previously, in [26], Albert *et al.* proposed a model to describe embryonic pattern formation in the fruit fly Drosophila melanogaster. Their Boolean model consisted of 60 variables whose steady states were identified by manually solving a system of Boolean equations. After this, authors in [27] developed a strategy for efficiently identifying attractors of the network. To analyze the model, they first standardized their variables using the Boolean rules described in [26] by renaming them, i.e., SLP_*i*_ or wg_*i*_ to *x*_1_, ⋯ , *x*_60_. The variable *x_i_* and corresponding genes are summarized in Table 4. They next applied their proposed method and obtained the steady states shown in Table 5. Each row in Table 5 corresponds to a stable attractor and each column represents a gene (or protein). Attractors have been denoted as binary values with 1 representing a gene being expressed (or high protein concentration), and 0 representing a gene not being expressed (or low concentration). Based on the established given attractor set, we successfully applied our model to identify the minimum cardinality of contiguous nodes (i.e., 22) necessary to distinguish them. Specifically, (*x*_3_, ⋯ , *x*_24_) are the desired minimum consecutive variables which implies we can use these 22 nodes to discriminate the attractors and the 22 variables correspond to a list of genes or proteins which are shown in Table 6.

## V. CONCLUSION

In this work, we addressed a novel problem, observability of singleton or cyclic attractors, which is of value in distinguishing different attractor cycles. We have developed an effective and efficient methodology to solve the captured problem, and complexity analysis shows our methodology works in *O*(*P^m^n*) time. As the number of attractors (*m*) is typically small, our method can be employed to resolve large-scale networks in addition to medium-size ones. We have also performed computational experiments verifying the efficiency and effectiveness of our novel method, and applied our algorithm to characterize a real-world scenario, i.e., segment polarity gene expression in Drosophila melanogaster. Our results suggest that our strategy may provide a novel tool for use in identifying useful genetic network biomarkers for the detection of disease. In the future work, further investigations will focus on developing more efficient methods to solve the captured problem.

## Figures and Tables

**TABLE 1.** The truth table.

State	*υ*_1_(*t*)	*υ*_2_(*t*)	*υ*_3_(*t*)	*υ*_1_(*t* + 1)	*υ*_2_(*t* + 1)	*υ*_3_(*t* + 1)
1	0	0	0	1	1	0
2	0	0	1	1	1	0
3	0	1	0	0	1	0
4	0	1	1	0	1	0
5	1	0	0	1	0	1
6	1	0	1	1	1	1
7	1	1	0	0	0	1
8	1	1	1	0	1	1

**TABLE 2.** Results on observability of singleton attractor for our method.

*n/m*	100/10	150/15	200/20	250/25	300/30
Time (sec)	0.013	0.02	0.024	0.029	0.047
numNode	4.1	5.1	6	6.5	6.9

**TABLE 3.** Results on observability of cyclic attractors with period 2.

*n/m*	100/4	400/5	600/6	800/7	1000/8
Time (sec)	0.19	0.19	0.15	0.75	1.42
numNode	2	2.1	3	3	3.2

**TABLE 4.** Correspondence of variables and gene names.

compartment 1	SLP *x*_1_	wg *x*_2_	WG *x*_3_	en *x*_4_	EN *x*_5_	hh *x*_6_	HH *x*_7_	ptc *x*_8_	PTC *x*_9_	PH *x*_10_	SMO *x*_11_	ci *x*_12_	CI *x*_13_	CIA *x*_14_	CIR *x*_15_
compartment 2	SLP *x*_16_	wg *x*_17_	WG *x*_18_	en *x*_19_	EN *x*_20_	hh *x*_21_	HH *x*_22_	ptc *x*_23_	PTC *x*_24_	PH *x*_25_	SMO *x*_26_	ci *x*_27_	CI *x*_28_	CIA *x*_29_	CIR *x*_30_
compartment 3	SLP *x*_31_	wg *x*_32_	WG *x*_33_	en *x*_34_	EN *x*_35_	hh *x*_36_	HH *x*_37_	ptc *x*_38_	PTC *x*_39_	PH *x*_40_	SMO *x*_41_	ci *x*_42_	CI *x*_43_	CIA *x*_44_	CIR *x*_45_
compartment 4	SLP *x*_46_	wg *x*_47_	WG *x*_48_	en *x*_49_	EN *x*_50_	hh *x*_51_	HH *x*_52_	ptc *x*_53_	PTC *x*_54_	PH *x*_55_	SMO *x*_56_	ci *x*_57_	CI *x*_58_	CIA *x*_59_	CIR *x*_60_

**TABLE 5.** Steady states of a drosophila model.

000000001001101000000001001101100000001001101100000001001101
000111100010000000111100010000111000011111110111000011111110
000000011111110000111100010000111000011111110100000001001101
011000011111110000111100010000111000011111110100000001001101
000000011111110000111101000000111000011111110100000001001101
011000011111110000111101000000111000011111110100000001001101
000111100010000000000011111110100000001001101111000011111110
000111101000000000000011111110100000001001101111000011111110
000111100010000011000011111110100000001001101111000011111110
000111101000000011000011111110100000001001101111000011111110

**TABLE 6.** detected minimum 22 genes or proteins.

compartment 1	WG,en,EN,hh,HH,ptc,PTC,PH,SMO,ci,CI,CIA,CIR
compartment 2	SLP,wg,WG,en,EN,hh,HH,ptc,PTC
